# Purulent Pleurisy

**Published:** 1906-06-09

**Authors:** 


### PURULENT PLEURISY.

Purulent pleurisy is a lesion of exceptional interest on account of the difficulty which may attend its identification and the gratifying nature of the results which follow its discovery and correct treatment. Before proceeding to review the main features of the malady, it may be well to define what is meant by purulent pleurisy or empyema, for there are occasions when pleural exudations present to the naked eye characters so ambiguous as to leave room for doubt whether they should be considered purulent or not. It must suffice at present to say that the unaided eye is often incapable of forming an accurate judgment on the matter. Whenever an effusion recovered from the chest departs from the characteristic type of the serous effusion proper, it should be submitted to microscopical examination after having been suitably stained to show any micro-organisms which may be present. If under these circumstances leucocytes be found in any abundance, it is probable that the field will contain also considerable numbers of micro-organisms. Any fluid which contains micro-organisms in quantity has good title to be considered pus, even though the leucocytes present are so few as to leave it lacking in the milkiness typical of pus.

Ætiologically an empyema depends upon the infection of the pleura by micro-organisms. The organisms capable of producing the lesion are many in number, and the clinical type of the malady will vary to a considerable extent with the nature of the causative parasite. But the organism of most frequent occurrence is the pneumococcus. Relatively to pneumococcal empyemata all other varieties are rarities, although not absolutely uncommon. Next in frequency to the pneumococcus, as causes of empyema, come the ordinary pyogenic cocci, the staphylococcus pyogenes, both albus and aureus, and the streptococcus.

Empyema may be either primary or secondary—that is to say, either associated with a known antecedent lesion or not. In the case of pneumococcal empyemata the disease occurs most often as a sequel of lobar pneumonia, although it may appear, especially in childhood, after broncho-pneumonia, or even bronchitis. Empyemata due to other organisms are almost invariably secondary to some known lesion either in the lung or some distant part of the body. For purposes of clinical description it will be well to consider the disease in relation to its bacteriology, the pneumococcal forms having claims to be considered as a group apart, while the pyogenic or septic varieties have so many general features in common as to warrant their being dealt with together.

Pneumococcal empyemata may be either acute or chronic. Of these two varieties the chronic is by far the more frequently met with, and will first receive attention. The morbid anatomy of this disease consists in the collection of a pleural exudate having the following characters. It is a thick creamy fluid, greenish-yellow in colour, odourless, and often containing large masses of gelatinous purulent material. These gelatinous collections represent fibrinous coagula holding pus-cells in their meshes. Fibrin-formation is a characteristic function of the pneumococcus, so much so that the presence of fibrinous coagula in a purulent effusion in the pleural cavities is almost conclusive evidence of the action of this organism. The extent of surface covered by the effusion will depend, of course, chiefly upon the bulk of the latter; but not entirely, since the spread of the effusion over the lung is dictated by the presence or absence of adhesions. In consequence an empyema of little bulk may in the absence of limiting adhesions cover a large area of lung. As a matter of fact empyemata of any duration become delimitated by very dense adhesions, an important matter when the surgery of the malady comes to be considered. The commonest site for empyemata is the lower part of the chest, a selection enforced by gravity. But there is no area of the lung, be it never so small, which may not on occasion be the site of a small loculated collection of pus. Thus empyemata are occasionally apical, although confinement of the effusion to the extreme apex is probably an extreme rarity; or they may be entirely basal—that is to say, situated entirely between the under surface of the lung and the upper surface of the diaphragm; or lastly, they may be entirely interlobar.

The long continuance in the pleural cavity of a large collection of pus gives rise to pleural thickening of a degree not met with in other lesions. In addition the prolonged pressure of the exudate is capable of reducing the lung on the affected side to a mere knob of fibrous tissue. Purulent exudations of moderate size are certainly capable of spontaneous absorption. In such cases the fluid elements of the exudate are gradually absorbed, the solid ingredients assuming the appearance of a yellowish membrane upon the lung or parietes of chest. Such an inspissated collection of pus is prone to have deposited in it earthy salts which may convert the membrane into a calcerous plate. Clinically, as has been said, chronic pneumococcal empyemata occur most often as a sequel of lobar pneumonia, but cases are not infrequent when no such association can be established. There is no doubt that the lesion is occasionally primary. The type of an ordinary case is as follows: A patient undergoes a bout of frank lobar pneumonia. After a certain lapse of time the crisis occurs, and the patient appears to enter upon convalescence. But presently the temperature chart begins to show an irregular fever, which is accompanied by the development of signs of a fluid effusion in one side or other of the chest. Sometimes there may be no critical fall of temperature, but the fever from being continuous becomes intermittent or remittent. As time goes on, if the condition be not recognised, the patient begins to show signs of intoxication. He wastes, and commonly shows some clubbing of the fingers. In addition he develops an anæmia which gives a peculiar straw colour to the skin—a colour which in the presence of pulmonary symptoms is not a little suggestive of empyema. If no active treatment be adopted the disease proceeds to its conclusion along the following alternative routes. Either the patient emaciates progressively and dies from exhaustion, or the purulent collection is spontaneously discharged. This may be achieved either by inspissation in the fashion mentioned above, or by the rupture of the abscess. Rupture again may take place either externally through an intercostal space, or internally through an eroded air-tube. In the latter case the whole abscess may be expectorated and cure result after a long and tedious illness.

The acute variety of pneumococcal empyema is generally an expression of a blood-infection by the pneumococcus, a true septicæmia manifesting itself locally. In these cases the exudation is, to the naked eye, of the ambiguous nature described above. It appears as a slightly turbid serous fluid, inodorous, and sometimes, in very acute infections, stained with blood. Under the microscope this fluid is seen to carry in suspension a considerable number of leucocytes, and diplococci in varying abundance. Occasionally these organisms may be present in such quantity as to give the impression that a broth culture of the parasite is being contemplated. Such acute empyemata may form very rapidly, after a few days of vague ill-health, and attain considerable dimensions. The acuteness of the infection seems to inhibit the formation of fibrin so commonly seen in the operations of the pneumococcus, and loculation of the effusion is consequently rare.

Empyemata due to organisms other than the pneumococcus generally indicate some local or general sepsis. The characters of the pus in such cases is not distinctive of the infecting organism, but the greenish-yellow colour of pneumococcal pus is seldom simulated. The commonest cause of such empyemata is some septic focus within the lung, whether infarction by an infective embolus as may happen during osteo-myelitis or infective thrombosis of the systemic veins, or abscess within the lung, or spread of infection from a secondarily-infected tuberculous cavity. In either of these latter cases actual rupture of the offending focus may convert the empyema into a pyo-pneumothorax. In such cases the presence of putrefactive organisms often gives to the pus an extremely offensive odour. The course of such empyemata will be dictated by the nature and gravity of the primary lesion.

The diagnosis of empyema may be a matter of the greatest ease or almost insuperable difficulty. In a straightforward case, where the bulk of the effusion is considerable, the existence of a fluid effusion is not in doubt, and its purulent nature is easily demonstrated by means of an exploring needle. It may be worth while, however, to point out certain fallacies which attend the use of the needle in practice. Attention has already been drawn to the thick consistency of pneumococcal pus, and the tendency evinced by the pneumococcus towards the formation of fibrin. These characters of the pus render blocking of the needle a frequent occurrence. It is important, therefore, that a negative result upon exploration of the chest should not be regarded as conclusive evidence of the absence of pus, for it may be necessary to puncture the chest in several places before pus is discovered. A practical hint of some value is the following manœuvre:—When the needle is withdrawn after a fruitless effort, the contents of the bore of the needle should be expressed upon the thumb-nail of the left hand. In this way it is possible to discover whether or not the failure has been due to the presence of thickened pus or flakes of fibrin. A second fallacy of less importance depends upon the fact that the degeneration of a caseous pneumonia may give rise to a large tuberculous intra-pulmonary abscess. In such a case exploration may result in the finding of pus and the consequent assumption that the lesion is empyema, whereas it is in fact one of far greater gravity.

It is on occasions when the empyema is small that detection becomes so difficult a matter, a difficulty which will be enhanced when the effusion is situated between the lobes of the lung or between the lung and the diaphragm. In such circumstances all the resources of medicine may hardly suffice to achieve success. For the detection of an interlobar empyema the utmost nicety of physical examination will be required, since in such cases the effusion reaches the surface over a small area only, and one following the interlobar lines of the lung. It is essential, therefore, to remember that the great fissure of the lung runs on either side from the level of the second dorsal spinous process behind in a curve with its convexity outwards to end in the region of the sixth costo-chondral joint. On the right side the secondary fissure between the upper and middle lobes leaves the main fissure in the axillary line and runs forwards and slightly downwards towards the fourth rib-cartilage. When the empyema is diaphragmatic it may depress the liver if situated on the right side. It is well to bear in mind that, since the dullness of the percussion-note over a fluid effusion depends more upon the condition of the underlying lung than upon the mere damping effect of the effusion itself, a considerable effusion may coexist, especially in young subjects, with a fair percussion note, and also with loud bronchopony and bronchial breathing. The use of the exploring needle must never be omitted, while both radiography and an absolute count of the leucocytes in any given case may render valuable aid in diagnosis. Much has been written of late concerning the dangers of exploratory puncture of the chest. Certainly the puncture of a solid and inflamed lung is to be deprecated, for alarming hæmorrhage may follow such a proceeding. Extensive surgical emphysema is also a rare and occasional consequence. But fears of such rare misadventures must not be given too great a weight. If a patient suspected of a localised empyema be spontaneously improving, the academic success of pus discovery is a trifle of no importance. But if the patient be steadily losing ground, perseverance in the use of the needle becomes a duty.

The prognosis of empyema may be said to vary with its bacteriology. When the disease depends upon the pneumococcus, and is of the common chronic variety, the outlook is good, provided that the abscess be freely drained. The acute variety of this lesion, being often an expression of septicæmia, is always grave and often fatal in spite of treatment. Empyemata due to pyogenic cocci always constitute a serious disease, for they indicate, as we have said, a septic process in the lung or elsewhere. The outlook, in consequence, is largely determined by the nature of the primary lesion.

The end of treatment in the case of empyema is, of course, the evacuation of the abscess. The alternative methods are three—namely, aspiration, simple incision of the pleura through an intercostal space, or excision of a portion of rib preliminary to the opening of the pleura. There is no doubt that empyemata consisting of thin pus may occasionally be cured by aspiration, but the method does not find general acceptance. Between the other two alternatives there is not in most cases much to choose. It may be said, however, that in the case of very young children simple incision is preferable to the other, for it may be readily undertaken under local anæsthesia. If a general anæsthetic be employed for the operation upon empyema the cardinal rule of safety is to give the smallest quantity which will produce insensibility.

